# Quality of antenatal care services in Rwanda: assessing practices of health care providers

**DOI:** 10.1186/s12913-018-3694-5

**Published:** 2018-11-19

**Authors:** Akashi Andrew Rurangirwa, Ingrid Mogren, Joseph Ntaganira, Kaymarlin Govender, Gunilla Krantz

**Affiliations:** 1 Department of Epidemiology and Biostatistics, School of Public Health, University of Rwanda, Kigali, Rwanda; 2 Section of Epidemiology and Social Medicine (EPSO), Department of Public Health and Community Medicine, The Sahlgrenska Academy at University of Gothenburg, Gothenburg, Sweden; 3 Department of Clinical Sciences, Obstetrics and Gynecology, Umeå University, Box 453, 405 30 Göteborg, Sweden; 4 Judith Lumley Centre, La Trobe University, Melbourne, Australia; 5 Health Economics and HIV/AIDS Research Division (HEARD), University of KwaZulu-Natal, KwaZulu-Natal, 4000 South Africa

**Keywords:** Antenatal care, Quality of care, Antenatal care providers, Practices, Rwanda

## Abstract

**Background:**

Although most pregnant women in Rwanda visit antenatal care (ANC) clinics, little has been studied about the quality of services being provided. We investigated the ANC providers’ (HCPs) current practices in relation to prevention, management and referral of maternal conditions as well as the information provided to pregnant women attending ANC services in Rwanda.

**Methods:**

This facility-based, cross-sectional study included 312 ANC providers as participants and a review of 605 ANC medical records from 121 health centers. Data collection was performed using an interviewer-administered questionnaire and a structured observation checklist. For the analyses, descriptive statistics and bi-and multivariable logistic regression were used.

**Results:**

Nurses and midwives in ANC services failed to report a number of pregnancy-related conditions that would need urgent referral to a higher level of health care. Midwives did somewhat better than nurses in reporting these conditions. There was no statistically significant difference in how nurses and midwives informed pregnant women about pregnancy-related issues. Ever been trained in how to manage a pregnant woman exposed to violence was reported by 14% of the participants. In 12, 13 and 15% of the medical records there was no report on tetanus immunization, anthelmintic treatment and syphilis testing, respectively.

**Conclusion:**

The providers in ANC clinics reported suboptimal practices on conditions of pregnancy that needed urgent referral for adequate management. Information to pregnant women on danger signs of pregnancy, recommended medicines and tests do not seem to be consistently provided. Midwifery training in Rwanda should be expanded so that most of staff at ANC clinics are trained as midwives to help lower maternal and child mortality and morbidity.

**Electronic supplementary material:**

The online version of this article (10.1186/s12913-018-3694-5) contains supplementary material, which is available to authorized users.

## Background

Antenatal care (ANC) offers a superb opportunity to inform and educate pregnant women on important health issues including health promotion activities, screening and diagnosis [1]. ANC is also an important platform to communicate with and support women, families and communities at a critical time in a woman’s life. This is especially important for sensitive health matters (e.g. intimate partner violence, IPV) that may otherwise be overlooked. It is known that violence during pregnancy may endanger maternal and fetal health, as well as mental health [2]. The perpetrator is usually the husband or partner and we have previously shown that partner violence is common and may increase during pregnancy among Rwandan women [3]. Furthermore, ANC attendance increases the chances of better management and outcome of the most common complications (e.g. infection, pregnancy-induced hypertensive disorders and severe bleeding) associated with maternal morbidity and mortality in low-income countries [4, 5]. Identifying pregnant women at risk and timely referral practices are known to reduce maternal and neonatal morbidity and mortality [6–8]. Thus, ANC is an essential component of the Birth Preparedness and Complications Readiness (BPCR) matrix that encompasses all the responsibilities, actions, practices and skills needed to ensure the safety and well-being of the pregnant woman and her newborn throughout pregnancy, labour, childbirth and postpartum period [9].

Since 2002, there has been a progressive increase in the use of ANC services among pregnant women in low and middle-income countries, leading to marked improvements in maternal and child health [1, 10–12]. Nevertheless, maternal and perinatal mortality remains high [13], suggesting that there is still a need to improve the quality of care given in the ANC clinics and during delivery. ANC services should be provided by skilled health professionals who can identify inter-current diseases of significance and risk factors associated with pregnancy and childbirth complications. Of particular importance is to provide relevant education and counseling, to identify signs of the most dangerous conditions and formulate a delivery plan such that emergency situations can be avoided or handled successfully [1]. Studies from low-income countries have revealed significant gaps and weaknesses in knowledge and practices of ANC providers regarding provision of essential elements of the ANC package including counseling pregnant women on danger signs [14, 15]. If women and their families were thoroughly informed about danger signs and advised to seek health care immediately if any occurs, maternal morbidity and mortality could be reduced dramatically [16].

ANC services in Rwanda are typically provided at health centers with district hospitals as the first referral units [17]. District hospitals are generally well equipped and have the capacity to perform surgical procedures, but more serious cases are referred to the national referral hospitals [17]. The maternal mortality ratio has been improving after the devastating effects on the country’s health infrastructure during the 1994 Genocide against the Tutsi. This improvement is mainly due to increased attendance at ANC services and that more women today are delivering at a health facility [3, 18]. However, maternal and neonatal mortality rates remain high: 210 maternal deaths per 100,000 live births and 20 neonatal deaths per 1000 live births [12]. Thus, access to quality ANC services is crucial to achieve the 2030 agenda for Sustainable Development Goals (SDGs). Little is known about the practices of ANC providers in Rwanda (of which most are nurses). Available data, however, suggest that inadequate care stemming from a lack of knowledge and skills to perform interventions or counsel women on important aspects of maternal and neonatal health may be an issue [19, 20].

Therefore, we investigated the current ANC providers’ practices in prevention, detection, management and referral of maternal conditions. Furthermore, the occurrence of violence during pregnancy and the information and services provided to pregnant women attending an ANC facility were investigated. This study is part of the Maternal Health Research Programme (MaTHeR) undertaken by the University of Rwanda in collaboration with the University of Gothenburg and Umeå University in Sweden.

## Methods

### Study design, study population and sample size

This facility-based cross-sectional study was conducted at health centers (*n* = 121) in the Northern Province of Rwanda and Kigali city. Kigali city has urban, semi-urban and rural areas, whereas the Northern Province of Rwanda is predominantly rural. The study area has been described in detail elsewhere [18]. All 121 health centers in the study area were included in the study and the number of ANC providers to be interviewed from each facility was selected based on the total number of the providers employed at each health center (proportionate to size). In almost all health centers, no providers are assigned to specifically work in ANC clinics; they rotate to all services in the health center as per duty roster. The sample size was calculated according to the total population of health care providers (*n* = 1890, nurses and midwives) employed at the health centers in the study area. Using the formula; sample size = n / [1 + (n/population)] where n = Z * Z [P (1-P)/(D*D)], P = the expected frequency value of ANC providers with poor knowledge of pregnancy conditions calling for urgent assessment at a higher level of care (assumed to be 50%), D = the acceptable margin of error (5%) and Z = area under normal curve corresponding to the desired confidence level (95%/1.960), the sample size was calculated to 319 participants [21]. Because, we restricted our study to the providers who had ever attended to a pregnant woman in any ANC clinic before the day of the interview, seven providers were excluded. Thus, the final sample consisted of 312 ANC providers from the 121 health centers. In collaboration with the administration at the health centers eligible providers were identified and asked to participate. A random selection by drawing numbers from the table was performed if an excess number of eligible providers were available at any of the health facilities.

Concurrently and in the same health facilities, a sample of ANC medical records was reviewed, with the aim to identify key services that were offered to pregnant women during pregnancy. Using the WHO’s sampling scheme for ANC medical records review, 605 medical records were needed [22]. A systematic random sampling was applied to obtain the five ANC medical records that were required from each health center. First, the total number of ANC medical records containing at least one visit by a pregnant woman in the past 12 months at each health facility was determined. Second, the available number of ANC medical records was divided by five and a sampling interval was obtained. Finally, one ANC record was pulled at each interval. The analysis in this study was restricted to records with ≥3 visits (i.e. 233 records).

### Data collection procedures

Data collection took place between October and December 2015. A structured, paper-based interviewer-administered questionnaire (Additional file 1: Q1) was developed for the interview study. The questionnaire items measured education and training in the delivery of ANC services. The items also evaluated ANC providers’ practices related to conditions needing urgent assessment at a higher-level health facility during pregnancy. The questionnaire further contained items about what general information was given to pregnant women attending ANC clinics, including assessment and management of IPV during pregnancy. The items on providers’ practices were selected based on the WHO manual for implementation of ANC services provision that has been adopted by the Ministry of Health in Rwanda [23]. The questionnaire was translated into Kinyarwanda, the Rwandan national language, and pre-tested on 12 ANC providers in the Eastern province. No major changes were made in the questionnaire after the pre-test, apart from a few adjustments in Kinyarwanda wording.

For the interview study, four well-trained interviewers (all were registered nurses) belonging to a pool of data collectors at the School of Public Health, University of Rwanda conducted face-to-face interviews with the providers, with guidance from two supervisors (first and third authors). All the interviews, ranging from 45 to 60 min, took place in the ANC consultation room. If it was not possible to conduct the interview with the selected provider at a certain time for any given reason, the interview was arranged another time. No ANC provider refused to participate in the interview. The School of Public Health at the University of Rwanda was the lead facilitator of the study. Data entry was performed by a skilled data entry clerk, selected from a permanent cohort at the University of Rwanda’s School of Public Health under the supervision of a data entry manager. After primary data entry, information from 20 randomly selected questionnaires was re-entered by the first author to check the accuracy of the first data entry. No erroneous entries were detected.

The items for the review of pregnant women’s ANC medical records were adopted from two sources: the WHO Safe Motherhood Assessment Manual and Rwanda’s Ministry of Health prenatal monitoring recommendations [22]. A pre-tested, structured observation checklist (Additional file 1: Q1) was used to record whether specific recommended procedures were registered. The procedures on the checklist included provision of tetanus immunization, syphilis test, anthelminthic treatment and malarial prevention by providing insecticide-treated nets to pregnant women.

### Variables

#### Conditions needing urgent assessment at a higher-level health facility

ANC providers were asked to mention any urgent pregnancy-related condition that would need prompt referral of a woman to a hospital for more advanced management. This was an open question, where the providers were encouraged to mention as many patient conditions as they could recall. Based on the WHO and Rwanda ANC services recommendations for better pregnancy outcome, nine conditions were selected as needing urgent referral to a facility offering more advanced management: severe hypertension, fits, cessation of fetal movements, preeclampsia, heavy vaginal bleeding, severe abdominal pain, visual disturbances, previous C-section and diabetes mellitus [1, 23]. Subsequently, a summary measure that characterized the providers’ mentioning of the nine conditions was created, with scores ranging from 0 to 9. The summary measure variable was later dichotomized and used as the outcome variable in assessing the factors that would be associated with poor performance.

#### Intimate Partner Violence

ANC providers were asked i) whether they had encountered a pregnant woman in the clinic who had been exposed to any form of IPV*,* ii) whether they had ever been trained in how to take care of a pregnant woman who had been exposed to any form of violence (physical, sexual and/or psychological) and iii) what they would do if a pregnant woman told them that she has been exposed to any violent acts during pregnancy. The first two were *yes/no questions.* The third question had five response choices (all with *yes/no* response alternatives).

#### Socio-demographic and psychosocial variables

*Participants’ age* was categorized into three age groups: 21–30, 31–40 and ≥ 41 years. *The ANC providers’ highest attained education level was* described as a three-category variable i.e. A2 nurses, A1/A0 nurses and A1/A0 midwives. There are three different levels of competence in health care providers’ training in Rwanda who are eligible for ANC employment i.e. A2 nurses, A1 nurses or midwives and A0 nurses or midwives. A2 level nurses have six years of secondary school training with a general nursing focus. Some of A2 nurses were A3 nurses (auxiliary nurses with three years of post-primary school training in nursing) who later continued their education and were upgraded to A2 nursing level. A1 nurses have continued with three years at an institution of higher education while A0 nurses completed four years obtaining a qualification equivalent to a Bachelor of Science Degree. A1 and A0 are registered nurses (RNs). A1 and A0 midwives completed 3 and 4 years of training in midwifery at an institution of higher education, respectively. Although training of A2 nurses in Rwanda ended in 2007, most practicing nurses are still at this level. *Participants’ work experience in ANC services* was dichotomized into ≥4 years or ≤ 3 years, with the latter serving as the exposure category. *Ever attended ANC services delivery training* was dichotomized as *yes/no* responses with the latter functioning as the exposure category. *Consultation time* was first reported and analyzed as a continuous variable (minutes) and later dichotomized into the categories ≥16 min and ≤ 15 min, with the second category as the exposure category. The items from the ANC medical records were responded to with *yes* if the item was recorded in the register and *no* if it was not.

### Statistical analysis

Data were analyzed descriptively using frequencies and percentages to describe ANC providers’ characteristics, information given to pregnant women and analyses pertaining to violence during pregnancy. Similar procedures were used to describe the providers mentioning of conditions needing urgent assessment at a higher-level health facility and procedures that were or were not recorded during visits made to the ANC clinics. Associations between participant demographic characteristics (predictor variables) and mentioning of conditions needing urgent assessment during pregnancy (outcome variable) were investigated using bivariable and multivariable logistic regression models. Variables included in the models were determined based on their strength in the bivariable analysis and theoretical reasons grounded in previous research. Using this approach, six variables were retained in the final model. If two variables were highly correlated (*r* ≥ 0.40), one would be excluded. All measures of association are presented as odds ratios (ORs) with their 95% confidence intervals (95%CIs). All the analyses were performed using Statistical Package of Social Sciences version 23.0 for Windows (SPSS, Armonk, NY, USA).

### Ethical considerations

Participation was voluntary and no incentive was given for participating in the study. Before the interview, the interviewer explained the content of the questionnaire, informed the participants on confidentiality of their responses and that they could withdraw from the study at any time without consequences. The interview took place in privacy in the ANC consultation room. A written and signed consent was obtained from all participants*.* The data sets were anonymous with no possibility to identify the providers or individuals on the ANC medical records.

## Results

### Socio-demographic and psychosocial characteristics

Most participants (69.3%, *n* = 210) were A3/A2 nurses (Table 1). Only 7.9% (*n* = 24) of the participants were midwives. Just over two thirds had > 4 years of work experience and 7.9% (n = 24) had < 1 year of work experience in ANC services. Most participants (90%, *n* = 280) had not received any in-service training in the delivery of ANC services over the past 2 years and over half had never received any in-service training. The median patient-provider interaction time was 30 min (95% range: 9.5–60) and 25.3% (*n* = 79) of the participants spent an average of ≤15 min providing ANC services to a pregnant women.

**Table 1:** Participant characteristics (*N* = 312)

Variables	Frequency	Percent
Province (*n* = 312)		
Kigali	113	36.2
Northern province	199	63.8
Sex (*n* = 311)		
Female	245	78.8
Male	66	21.2
Age group (*n* = 308)		
20–30	90	29.2
31–41	168	54.5
≥ 42	50	16.2
Health care providers’ highest attained education level (*n* = 303)		
A3/A2 nurses	210	69.3
A1/A0 nurses	69	22.8
A1/A0 midwives	24	7.9
Years of work experience (*n* = 309)		
< 1 year	24	7.9
1–3 years	75	24.2
4–6 years	59	19.0
> 6 years	151	48.9
In-service training sessions in ANC^a^ services in the past 2 years (*n* = 310)		
No	280	90.0
Yes	30	10.0
Last time received training in ANC (*n* = 311)		
Never received training in ANC	172	55.5
Do not remember	12	3.8
≤ 1 year ago	17	5.8
2-5 years ago	82	26.6
> 5 years ago	28	8.9
Feedback on your work performance from your superior or colleagues (*n* = 312)		
No	19	6.1
Yes	293	93.9
Number of feedbacks during the past year (*n* = 292)		
1	39	13.4
2	23	7.9
3	45	15.4
≥ 4	185	63.4
Did you contact the CHW^b^ in the village to ask her to send a woman to ANC clinic (*n* = 310)		
No	45	14.5
Yes	265	85.5
Average duration of ANC consultation (minutes) (*n* = 309)		
≤ 15	79	25.3
≥ 16	230	73.7

^a^*ANC* antenatal care, ^b^*CHW* community health worker

### Advice and information given to pregnant women attending ANC clinics

Tables 2 and 3 summarize the information and advice that the ANC providers gave to pregnant women at the ANC clinics. Most of the providers provided advice and information on HIV/AIDS, diet and nutrition and those conditions demanding acute assessment during pregnancy. However, 20.9% (*n* = 63) of the providers did not discuss what to do if the pregnant women experienced problems requiring immediate attention such as fits or heavy vaginal bleeding. In addition, over one third of the providers did not discuss the place of birth. Forty-four percent (*n* = 133) of the ANC providers did not provide the pregnant women information on how to take care for the newborn child. Whether the providers inquired about the occurrence of violence during pregnancy was investigated and what measures were taken when such cases were identified. The study shows that only 14% (*n* = 44) of the providers had ever been trained in how to treat and support pregnant women exposed to violence and only 7.3% gave advice on exposure to violence during or before pregnancy. Offering advice and information was more often reported by midwives than nurses.

**Table 2:** Advice and information given to pregnant women attending ANC clinics by level of education of health care providers (*N* = 303)

	Total *N* = 303,	Nurse A2 *n* = 210,	Nurse A1 + A0 *n* = 69,	Midwives *n* = 24,
Advice and information	*n* (%)	*n* (%) with in group	*n* (%) with in group	*n* (%) with in group
Diet and nutrition				
No	15 (5.0)	12 (5.7)	2 (2.9)	1 (4.2)
Yes	287 (95.0)	197 (94.3)	67 (97.1)	23 (95.8)
Discuss the place of delivery				
No	102 (33.8)	73 (34.9)	22 (31.9)	7 (29.2)
Yes	200 (66.2)	136 (65.1)	47 (68.1)	17 (70.8)
Discuss what to do if a severe problem arises				
No	63 (20.9)	50 (23.9)	10 (14.5)	3 (12.5)
Yes	239 (79.1)	159 (76.1)	59 (85.5)	21 (87.5)
Discuss child spacing or family planning (contraceptives)				
No	92 (30.5)	72 (34.4)	15 (21.7)	5 (20.8)
Yes	210 (69.5)	137 (65.6)	54 (78.3)	19 (79.2)
Discuss sexually transmitted diseases and HIV/AIDS				
No	67 (22.2)	45 (21.5)	17 (24.6)	5 (20.8)
Yes	235 (77.8)	164 (78.5)	52 (75.4)	19 (79.2)
Give advice and information on how to take care of the newborn				
No	133 (44.0)	95 (45.5)	28 (40.6)	10 (41.7)
Yes	169 (56.0)	114 (54.5)	41 (59.4)	14 (58.3)
Give advice on exposure to violence during or before pregnancy				
No	280 (92.7)	191 (91.4)	65 (94.2)	24 (100.0)
Yes	22 (7.3)	18 (8.6)	4 (5.8)	0 (0.0)

**Table 3:** Intimate partner violence (IPV) discussed at ANC clinic visits (*N* = 312)

Violence during pregnancy	Frequency	Percent
Ever encountered a pregnant woman in the clinic exposed to IPV		
No	202	65.6
Yes	105	34.4
Ever encountered a woman in ANC exposed to violence from any family member or a friend		
No	236	76.4
Yes	73	23.6
Ever been trained in how to take care of a pregnant woman who has been exposed to violence		
No	267	85.9
Yes	44	14.1
What would you do if a pregnant woman tells you that she is exposed to violence?		
Talk to her and let her tell her story		
No	8	2.6
Yes	303	97.4
Ask her to come back within 1–2 weeks		
No	298	95.8
Yes	13	4.2
Refer her to a district hospital		
No	197	63.3
Yes	114	36.7
Advise her to seek care at a shelter (One-stop clinic)		
No	268	86.2
Yes	43	13.8
Suggest that she brings her husband/partner to the next visit		
No	252	81.0
Yes	59	19.0

### Conditions needing urgent assessment at a higher-level health facility and associated factors

**Fig. 1:**
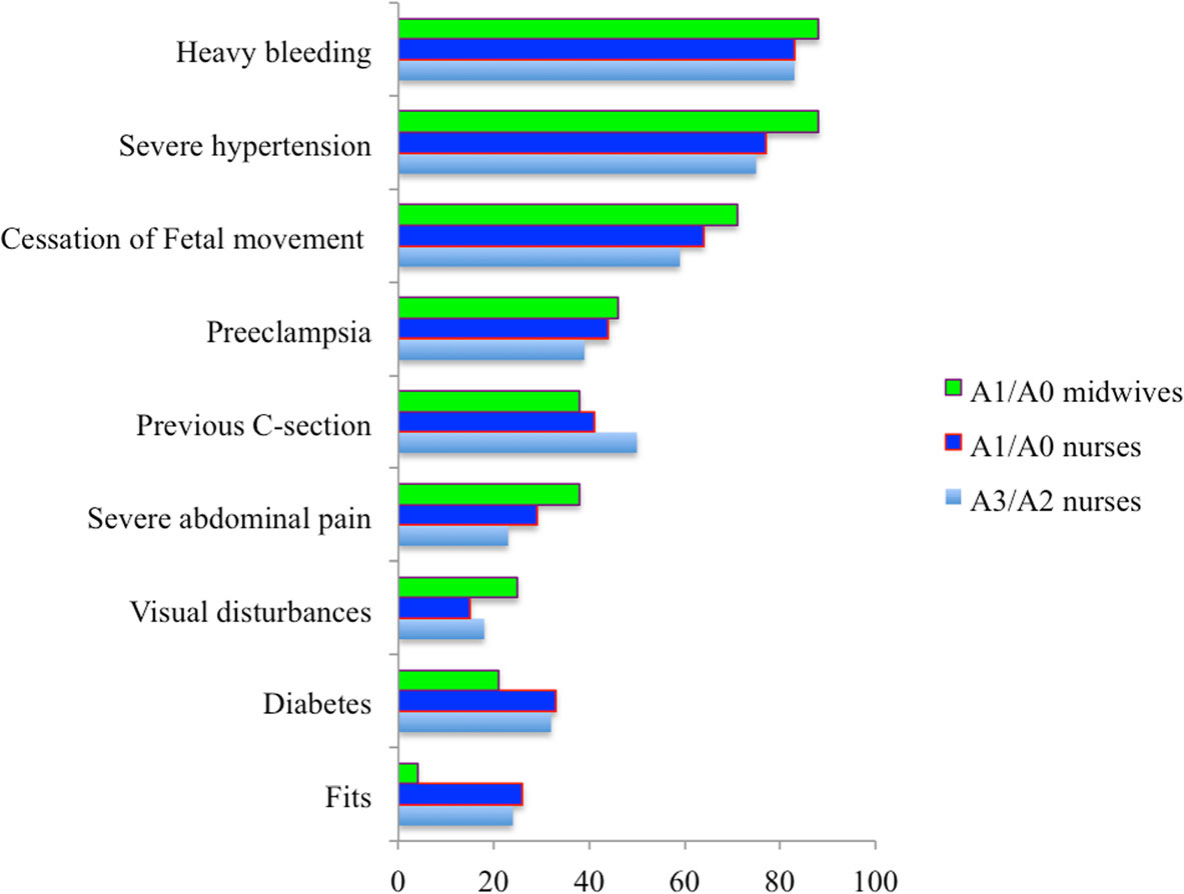
Providers’ mentioning of conditions requiring urgent assessment at a higher level of health care by providers’ level of education (values are percentages, *N* = 303)

**Fig. 2:**
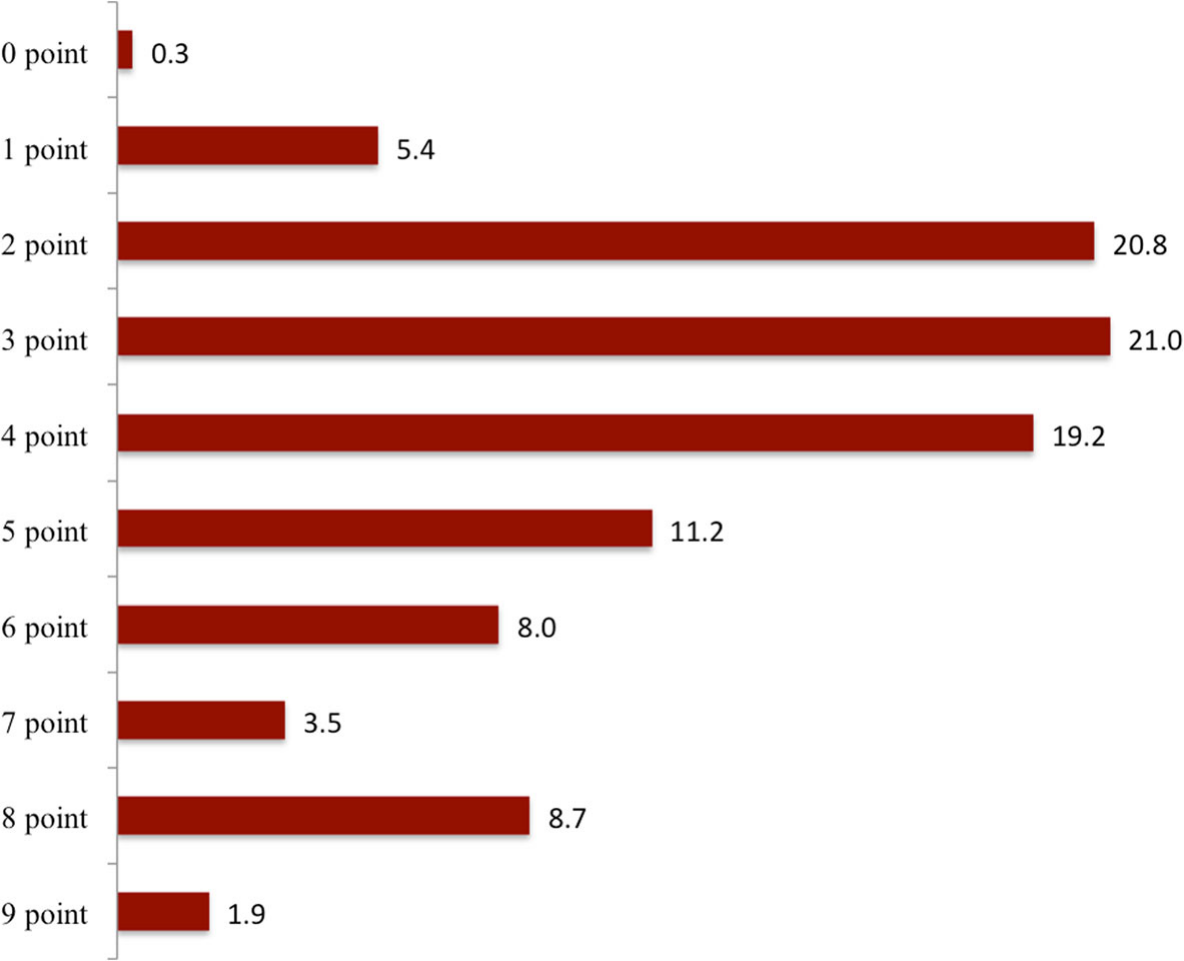
Summary measure of the providers’ mentioning of conditions requiring urgent assessment at a higher level of health care (values are percentages, *N* = 312)

One open question was designed to evaluate the providers’ ability to name the most dangerous conditions most likely to appear during pregnancy and labour and that would need prompt referral to a higher-level health facility for advanced surveillance and management. The following question was asked: “What symptoms and warning signs of pregnancy would make you promptly refer a pregnant woman to a hospital?” Figure 1 shows the proportions of the providers who mentioned any of the nine conditions considered to need urgent assessment at a higher-level health facility as a function of each educational level category (A3/A2 nurses, A1/A0 nurses and A1/A0 midwives). Most of the providers, regardless of educational category, mentioned heavy bleeding, severe hypertension and cessation of fetal movements. However, fits were mentioned by only 23% (*n* = 50) of the A3/A2 nurses, 26% (*n* = 18) of the A1/A0 nurses and 4% (n = 1) of the midwives. For preeclampsia, only 39% (*n* = 82) of the A3/A2 nurses, 44% (*n* = 30) of the A1/A0 nurses and 46% (*n* = 11) of the midwives mentioned this condition. Only 18% (*n* = 38), 15% (*n* = 10) and 25% (*n* = 6) of the A3/A2 nurses, A1/A0 nurses and midwives, respectively, mentioned visual disturbances. Proportion of the providers who mentioned conditions needing urgent assessment was somewhat higher among midwives than among nurses for most conditions, but none of these differences were statistically significant. Of all participants, only one (0.3%) did not mention any of the nine conditions and six (1.9%) mentioned all nine conditions (Fig. 2). Except for consultation time, which showed an adjusted OR of 2.14 (95% CI: 1.21, 3.78), there were no statistically significant associations of the providers’ socio-demographic and psychosocial characteristics with mentioning of few conditions (≤2 conditions). The exact estimates from the logistic regression analyses are given in Additional file 2: Table S1.

### Provision of essential elements of ANC

Of the pregnant women’s medical records investigated, 38.5% (*n* = 233) had ≥3 visits at an ANC clinic. Only these records were further analysed. Table 4 shows the recording of the recommended elements of ANC services in these case files. In 15% (*n* = 34) of pregnant women’s medical records syphilis testing was not recorded. Anthelmintic treatment was not reported in 13% of the records (*n* = 30) and tetanus immunization treatment in 12% (*n* = 28) of the records. Furthermore, 69% (*n* = 160) of the files did not record any information on malarial prevention by providing pregnant women with insecticide-treated mosquito nets.

**Table 4:** Recording of tests or a prescriptions in antenatal care medical files (*N* = 233)^a^

Test or prescription	Frequency	Percent
Haemoglobin^b^		
3rd visit		
No	167	71.7
Yes	66	28.3
4th visit		
No	198	85.0
Yes	35	15.0
Syphilis test		
No	34	15.0
Yes	199	85.0
Iron supplementation		
No	5	2.1
Yes	228	97.9
Malaria prophylaxis (insecticide-treated mosquito net)		
No	160	69.0
Yes	73	31.0
Tetanus Immunization		
No	28	12.1
Yes	205	87.9
Anthelmintic prophylaxis		
No	30	13.0
Yes	203	87.0
HIV test		
No	10	4.3
Yes	223	95.7
Proteinuria™		
3rd visit		
No	151	64.8
Yes	82	35.2
4th visit		
No	194	83.3
Yes	39	16.7

^a^Analysis was restricted to files with ≥3 visits. ^b^A laboratory test to be carried out if a pregnant woman shows physical signs of anemia. ™ test to be carried out during 3rd and 4th visit if a woman is nulliparous or has a history of hypertension, preeclampsia or eclampsia from a previous pregnancy

## Discussion

This study indicates that continuous training opportunities for ANC providers are still rare and that the information being given to pregnant women attending ANC services is not sufficient. Moreover, the practices of health care providers related to some maternal conditions requiring urgent assessment at a higher-level of care were suboptimal in this sample. Finally, some crucial elements of ANC services were not provided to all pregnant women.

Our findings show that 20.9% of the providers did not advise pregnant women on how to seek care if a danger sign or symptom needing prompt referral would arise. More than one third of the providers did not discuss the place of delivery with pregnant women and almost half did not provide information on how to take care of the newborn. These findings are consistent with those from other similar studies across the region [14, 24–26]. A multi-state study in Burkina Faso, Ghana and Tanzania demonstrated that one in three pregnant women was not given any information on any danger sign during pregnancy [24]. Similarly, counseling of pregnant women during ANC visits was found to be generally low in Haiti [27]. In Gambia, only 19.3% of pregnant women attending ANC clinics had been advised on what to do if there were a complication during pregnancy [25, 27]. The reason as to why ANC providers in Rwanda and other low-income countries may not use the opportunity to educate and advise pregnant women on important maternal and child health matters has thus far not been thoroughly investigated. However, it could be related to HCPs’ poor knowledge of the importance of counseling [28], or too little time that ANC providers spend with each pregnant woman because of understaffing [29]. The observation that some antenatal care providers may not be able to respond appropriately to pregnant women who have been exposed to violence is not surprising considering our results, which show that only 14% had ever been trained in how to handle such patients. Similar shortcomings have been reported from other African countries [30]. In line with related studies, we found that mentioning of maternal conditions needing urgent assessment was suboptimal for all educational levels of ANC providers. Similar findings have been reported from a Rwandan national health facility survey [19]. Studies from Uganda and Tanzania have shown that only 53.6% of ANC providers had adequate knowledge of ANC and only 6.1% of 115 providers answered correctly to all the questions related to knowledge and practices [31, 32]. ANC providers’ limited counseling of pregnant women on IPV during pregnancy and their inability to mention conditions needing urgent assessment at a higher health facility indicate a compelling need for training and refresher courses to reinforce and improve health care providers’ knowledge and skills. Furthermore, the observation in our study that, for most conditions, the proportions of providers who provided information to pregnant women and mentioning of conditions needing urgent assessment was higher among midwives than nurses is hardly puzzling. It emphasizes the need to put greater effort in educating and training more midwives in Rwanda [29]. The smaller number of providers across all educational categories who mentioned such conditions as fits, preeclampsia and visual disturbances is troubling. It may reflect that health care providers easily recall and erroneously perceive the conditions they most often observe in their practice as the most dangerous ones, but have insufficient knowledge on conditions, which are less commonly seen in their practice and therefore miss out on these.

We have shown that 25.3% of the ANC providers in Rwanda reported that they spent ≤15 min consulting a pregnant woman. This figure is lower than the recommendations of WHO and what has been observed in other countries [23, 33]. Although there is no conclusive evidence that spending more time with pregnant women would lead to better delivery of ANC services, our results suggest that those providers who spent ≥16 min were more likely to mention a larger number of pregnant conditions needing urgent assessment, suggesting that they spent more time examining pregnant women for these conditions and were therefore able to provide appropriate counseling. In agreement with another study in a low-income country, we found that some crucial elements of ANC services may not be provided [34]. Failure to carry out some of the procedures or provide some elements of ANC services package may be related to staff shortage or stock-outs of supplies [35].

### Methodological considerations

The strength of this study is that all the health centers in two of the five provinces in Rwanda took part in the study. Participants were recruited from all levels of education and randomly selected. Thus, we believe the findings are generalizable to ANC providers in the entire country. The sample size was large compared with other studies assessing health care providers’ knowledge and practices and all invited providers agreed to participate. However, the information inquired about was self-reported, which may have resulted in biased reporting (i.e. overestimation or underestimation of certain determinants). Furthermore, not all the tests, prescriptions and procedures that are recommended in the ANC package were assessed from ANC medical files. Also, it cannot be ruled out that some procedures or tests were done but the providers failed to record them in the files. However, this would constitute inadequate quality of care in relation to documentation procedures. We had a relatively small number of midwives in the sample (*n* = 24). Although this reflects the general picture of the shortage of midwives in the country [29], we still need to be circumspect in making any inferences about the variability between midwives and other ANC providers. Finally, although mentioning of conditions needing urgent assessment at a higher-level health care facility may not reflect the exact knowledge and skills of ANC providers, we believe that the ability to recall these conditions is a valid and reliable procedure to assess how providers perform during practice. A similar technique has been used to assess the knowledge of ANC providers [31].

## Conclusions

Although improvements have been made in maternal and child health in Rwanda, our results demonstrate that there are still serious deficiencies in ANC providers’ practices and medical record keeping. Accordingly, necessary measures should be taken to mobilize resources for education of more midwives to be stationed at ANC clinics and to aid in childbirth. Curriculum revision for courses leading to degrees in midwifery and nursing may be necessary to improve practices. Providers should be regularly trained and made aware of the importance of asking about violence exposure and other negative life circumstances that will affect a pregnant woman’s health status.

## Additional files

Additional file 1: **Q1.** Study questionnaire and observation checklist. (PDF 182 kb)

Additional file 2: **Table S1.** Associations between health care providers’ characteristics and poor knowledge of conditions needing urgent assessment at a higher level of health care facility during pregnancy. (DOCX 62 kb)

## Abbreviations

AIDS: Acquired immune deficiency syndrome
ANC: Antenatal care
AOR: Adjusted odds ratio
BPCR: Birth Preparedness and Complications Readiness
CI: Confidence interval
C-section: Cesarean section
DHS: Demographic and Health Survey
HIV: Human immunodeficiency virus
IPV: Intimate partner violence
MaTHeR: Maternal Health Research Programme
NISR: National institute of Statistics of Rwanda
SDGs: Sustainable development goals
WHO: World Health Organization
