# Supplementary material for: Quality of antenatal care services in Rwanda: assessing practices of health care providers
Source: BMC Health Serv Res. 2018 Nov 19;18:865. doi: 10.1186/s12913-018-3694-5 (PMC6245627; doi:10.1186/s12913-018-3694-5)
Supplement: Supplementary file 1 — Q1. Study questionnaire and observation checklist. (PDF 182 kb) [file 12913_2018_3694_MOESM1_ESM.pdf]

**ID.**

| | | |
|---|---|---|
| | | |

| |
|---|
| **A. SOCIAL-DEMOGRAPHIC INFORMATION INFORMATION** |
| **A1.** What is your<br>1. Province…………………………………………………….<br>2. District …………………………………………………….<br>3. District Hospital …………………………………………………….<br>4. Health center……………………………………………………. |
| **A2.** How old are you (Years)?………………….<br>**A3.** Sex |
| 1. ◯ Male |
| 2. ◯ Female |
| **A4.** Are you? |
| 1. ◯ Midwife? |
| 2. ◯ Nurse)? |
| 3. ◯ Auxiliary nurse? |
| 4. ◯ Other, specify. ……………………………… |

| **A5.** When was the last time that you attended to a pregnant woman at an ANC Clinic? **Tick one best response. If the respondent has never attended antenatal care client, thank her and close the interview** | |
|---|---|
| 1. ◯ | It has never happened |
| 2. ◯ | In the past week |
| 3. ◯ | In the past Month |
| 4. ◯ | In the past 6 months |
| 5. ◯ | 6 Months ago or longer |

| **A6.** How many years of experience in your current position? | |
|---|---|
| 1. ◯ | Less than 1 year |
| 2. ◯ | 1-3 years |
| 3. ◯ | 4-6 years |
| 4. ◯ | Over 6 years |

| **A7.** What is the highest level of education you have completed? |
|---|

| | |
|---|---|
| 1. ◯ | A2 (last 3years of secondary school in nursing sciences) |
| 2. ◯ | A1 A1 (3years of training after secondary school) |
| 3. ◯ | A0 (4years of training after secondary school) |
| 4. ◯ | Other, specify…………… |
| **B. IN-SERVICE TRAINING** | |
| **B1.a.** Have you been invited to any in-service training sessions in ANC services during the past 2 years? **(If no jump to B6)** | |
| 1. ◯ | Yes |
| 2. ◯ | No |
| **B1.b.** If yes, have you attended any in-service training sessions in ANC services during the past 2 years? **(If no jump to B6)** | |
| 1. ◯ | Yes |
| 2. ◯ | No, I have not attended any training |

| | |
|---|---|
| **B2.** How many times have you been to ANC in-service training in the past year (1 year ago now)? Number of times…................................ | |
| **B3**. What were the themes in the in-service training session/s that you have attended**? (More than one answer possible)** | |
| 1. ◯ | How to receive the client in ANC clinic and take history (a kind reception) |
| 2. ◯ | Physical examination of the client (Observe, e.g skin coloration, jaundice, gait, edema etc.. and taking the vital signs (measuring body temperature, BP, Heart rate, Respiratory rate….) |
| 3. ◯ | How to manage clients who are victims of sexual violence |
| 4. ◯ | How to manage ANC data |
| 5. ◯ | Focalized antenatal care (FANC) [How to sensitize women to continuously consult for ANC] |
| 6. ◯ | Warning signs of pregnancy complications |
| 7. ◯ | Family Planning |
| 8. ◯ | Sexually transmitted diseases (HIV/AIDS, syphilis, gonorrhea, chlamydia etc.) |
| 9. ◯ | Other, specify ……………….. |
| **B4.** Did the in–service training improve your ANC service delivery | |
| 1. ◯ | Yes |
| 2. ◯ | No |
| **B5.a.** When you received ANC training, did it include hands on practical training? | |
| 1. ◯ | Yes |
| 2. ◯ | No |
| **B5.b.** If yes, what kind of practical training?**( More than one response possible)** | |
| 1. ◯ | General assessment of pregnant woman) |

| | |
|---|---|
| 2. ◯ | Estimating gestational age from the fundal height measurement |
| 3. ◯ | Diagnosing pregnancy |
| 4. ◯ | Assessing the fetus (Position of the fetus, fetal heartbeats etc.) |
| 5. ◯ | Diagnosing disorders of pregnancy (High/Low Blood Pressure, Anemia etc.) |
| 6. ◯ | Other, specify………………………………….. |
| **B6.** When was the last time you received training in ANC? | |
| 1. ◯ | In the past week |
| 2. ◯ | In the past month |
| 3. ◯ | In the past 6 months |
| 4. ◯ | In the past year |
| 5. ◯ | In the past 5 years |
| 6. ◯ | 5 years ago or longer |
| 7. ◯ | Do not remember |
| 8. ◯ | I have never received training in ANC |
| **C. WORK FEEDBACK** | |
| **C1.a.** Do you receive feedback on your work performance from your superior or colleagues? | |
| 1. ◯ | Yes |
| 2. ◯ | No |
| **C1.b**. If yes, how many times have you received feedback on your work performance in the past year? | |
| 1. ◯ | Once |
| 2. ◯ | 2 times |
| 3. ◯ | 3 times |
| 4. ◯ | 4 times or more |
| **D. CLIENT INFORMATION AND EDUCATION MATERIAL FOR CLIENTs** | |
| **D1.a.** What type of client information, education and communication materials for antenatal care are available here? | |
| 1. ◯ | None |
| 2. ◯ | Posters |
| 3. ◯ | Cards |
| 4. ◯ | Take-home leaflets |
| 5. ◯ | Other,specify……………………… |
| **D1.b.** If available**,** what is the their content? | |
| 1. ◯ | On warning signs of complications of pregnancy |
| 2. ◯ | On Family planning |
| 3. ◯ | On Sexually transmitted diseases/HIV/AIDS |
| 4. ◯ | On postpartum and newborn care |
| 5. ◯ | On Breast Feeding |
| 6. ◯ | On Maternal and antenatal nutrition |

| | |
|---|---|
| 7. ◯ | Other, specify |
| **D2.** Are any antenatal care information, education and communication activities targeted at men? | |
| 1. ◯ | Yes, there are some specifically for men |
| 2. ◯ | No, there is none specifically for men |
| 3. ◯ | Yes, together with wife when he accompanies her to ANC clinic |
| 4. ◯ | Don't know |
| **E. ANC SERVICES** | |
| **E1.** How often are antenatal care services provided in this health center? | |
| 1. ◯ | Every day apart from weekends |
| 2. ◯ | 2 days per week |
| 3. ◯ | 3 days per week |
| 4. ◯ | 4 days or more per week |
| **E2.** Does it ever happen that you contact the CHW in the villages and ask him/her to send the pregnant women to the ANC clinic? | |
| 1. ◯ | Yes |
| 2. ◯ | No |
| **E3.** How many minutes on average does a consultation with a pregnant woman take?<br><br>Number minutes………………………….. | |
| **E4.a.** After an ANC consultation, do you tell the woman a date for the next visit to pay to the ANC clinic? (**If 1 or 6 jump to E.5**) | |
| 1. ◯ | Always tells her |
| 2. ◯ | Often |
| 3. ◯ | Sometimes |
| 4. ◯ | Never |
| 5. ◯ | We write the date on his card that she takes home |
| 6. ◯ | We tell her and write the date on his card that she takes home |
| **E.4.b.** If not always, when do you give the pregnant woman a set time for the next visit? | |
| 1. ◯ | When the woman asks for it |
| 2. ◯ | Only when I suspect a complicated pregnancy |
| 3. ◯ | If the woman had a complicated pregnancy earlier |
| 4. ◯ | When a woman has done 4 visits and has a normal pregnancy |
| 5. ◯ | Other reason, specify ……………………………………………… |
| **E.5**. How many visits should a pregnant woman at least pay to the ANC during the course of her pregnancy if it is normal and with no complications? | |
| 1. ◯ | 1 |
| 2. ◯ | 2 |
| 3. ◯ | 3 |
| 4. ◯ | 4 |
| 5. ◯ | 4 or more |
| 6. ◯ | I don't know |
| **E6.** What symptoms and warning signs of pregnancy would prompt you to refer a woman to a hospital? **Do not Read out list. You may tick more than one option/Tick as many responses as possible** | |
| 1. ◯ | Previous C-Section Scars |

| | |
|---|---|
| 2. ◯ | Previous stillbirth |
| 3. ◯ | Hypertension/Hypotension |
| 4. ◯ | Visual disturbances |
| 5. ◯ | Headache |
| 6. ◯ | Swelling |
| 7. ◯ | Fits |
| 8. ◯ | Fever |
| 9. ◯ | Severe Abdominal Pain |
| 10. ◯ | Anaemia |
| 11. ◯ | Pallor |
| 12. ◯ | Fatigue |
| 13. ◯ | Breathlessness |
| 14. ◯ | Cessation of fetal movements |
| 15. ◯ | Abnormal Lie/Position of Fetus |
| 16. ◯ | Foul smelling discharge |
| 17. ◯ | Light bleeding/spotting |
| 18. ◯ | Hemorrhage/Heavy bleeding |
| 19. ◯ | Multiple pregnancy/large abdomen |
| 20. ◯ | Exposure to violence |
| 21. ◯ | Pre-eclampsia |
| 22. ◯ | Nausea and vomiting |
| 23. ◯ | Diabetes |
| 24. ◯ | Diarrhoea |
| 25. ◯ | Other,specify………… |
| **E7.** What kind of advice and information do you give to pregnant women attending the ANC? **Do not Read out list. Tick as many responses as possible** | |
| 1. ◯ | Advice and information about diet and nutrition |
| 2. ◯ | Discuss the place of birth |
| 3. ◯ | Discuss with the woman what to do if there is a problem during pregnancy like bleeding convulsions or fits |
| 4. ◯ | Discus children spacing or family planning (contraceptives) |
| 5. ◯ | Discuss Sexually transmitted diseases and HIV/AIDS |
| 6. ◯ | Give advice and information on how to take care of the newborn |
| 7. ◯ | Discuss exposure to violence during the pregnancy or before |
| 8. ◯ | Other-specify |
| **F. VIOLENCE** | |
| **F1.** Have you ever encountered a pregnant woman in the clinic exposed to violence from her husband/partner? | |
| 1. ◯ | Yes |
| 2. ◯ | No |
| 3. ◯ | Don't Know/I don't remember |
| **F2.** Have you ever encountered a pregnant woman in the clinic exposed to violence from any other family member or friend? | |
| 1. ◯ | Yes |
| 2. ◯ | No |
| 3. ◯ | Don't Know/I don't remember |
| **F3.** Have you ever been trained in how to manage a pregnant woman who has been exposed to violence in | |

| | |
|---|---|
| the ANC clinic? | |
| 1. ◯ | Yes |
| 2. ◯ | No |
| 3. ◯ | Don't Know/I don't remember |
| **F4.** What would you do if a pregnant woman tells you that she is exposed to violence? **(Tick as many responses as mentioned)** | |
| 1. ◯ | Talk to her and let her tell her story |
| 2. ◯ | Ask her to come back within 1-2 weeks |
| 3. ◯ | Refer her to a district hospital |
| 4. ◯ | Advice her to seek care at a shelter (One Stop (One Stop clinic) |
| 5. ◯ | Suggest that she brings her husband/partner to the next visit |
| 6. ◯ | Suggest that she leaves her husband/partner |
| 7. ◯ | Any other advice ……………………………………….. |
| **F5**. Is parental or spousal consent required for any ANC visits for adolescents? | |
| 1. ◯ | Yes |
| 2. ◯ | No |
| 3. ◯ | Don't know |
| **G. EQUIPMENT** | |
| **G1.** Which equipment that you require for ANC is available? (**Read**, **Observe Tick)** | |
| 1. ◯ | Armoire |
| 2. ◯ | Balance pèse personne avec toise |
| 3 ◯ | Bassin réniforme (haricot) |
| 4 ◯ | Toise drawn on the wall |
| 5. ◯ | Bureau |
| 6. ◯ | Boite à coton |
| 7. ◯ | Mettre luban |
| 8. ◯ | Intebe (3) |
| 9. ◯ | Chariot de soins |
| 10. ◯ | Cupule inox |
| 11. ◯ | Détecteur de pouls fœtal et gel |
| 12. ◯ | Escabeau 2 marches |
| 13. ◯ | Kit DIU |
| 14. ◯ | Kit spéculum vaginal |
| 15. ◯ | Paravent |
| 16. ◯ | Pissette (2) |
| 17. ◯ | Seau à pédale |
| 18. ◯ | Stéthoscope |
| 19. ◯ | Stéthoscope de Pinard |
| 20. ◯ | Table d'examen gynécologique |
| 21. ◯ | Tambour à compresses |
| 22. ◯ | Tensiomètre |
| 23. ◯ | Plateau à instruments |
| 24. ◯ | Gravidometre |
| 25. ◯ | Other,specify……… |
| **H. WORK ENVIRONMENT AND OWN SKILLS** | |
| **H1**. In your opinion, what are the three greatest problems facing the ANC services in this facility? What do you think are the solutions for these problems? | |

| |
|---|

| Problems | Solutions |
|---|---|
| 1.Insufficient health workers | 1.Recrute more health workers |
| 2. Not enough required skills | 2. Regular training |
| 3. Lack of equipment | 3. Acquire more equipment |
| 4. Other, specify… | 4. Other, specify… |

| **H2.** Which are the 3 most important things that you need to improve as regards your own knowledge and skills? | |
|---|---|
| 1. ◯ | Further eduction |
| 2. ◯ | In- service training |
| 3. ◯ | Salary Increase |
| 4. ◯ | Other |

**ID**

| | | |
|---|---|---|
| | | |

**Separate temperate**

**A. CHECK LIST FOR ANC REGISTERS (Choose 5 Files randomly/Health center from all files of past one year)**

**B. SOCIAL-DEMOGRAPHIC INFORMATION INFORMATION**

**A1.** What is your

5. Province……………………………………………………….
6. District ……………………………………………………….
7. District Hospital …………………………………………………….
8. Health center…………………………………………………….

| **B.1.** How many ANC Visits are recorded?<br>Number of Recorded visits …………………… | |
|---|---|
| **B.2.** Is the expected date of delivery calculated? | |
| 1. ◯ | Yes |
| 2. ◯ | No |
| **B.3.** Is the gestational age at the first visit recorded? | |
| 1. ◯ | Yes |

| | |
|---|---|
| 2. ◯ | No |
| **B4** | What was the gestational age at the first vist<br>Number of weeks …………….. |
| **B.5.** Was maternal weight measured and recorded at each visit? | |
| 1. ◯ | During 1st visit |
| 2. ◯ | During 2nd visit |
| 3. ◯ | During 3rd visit |
| 4. ◯ | During 4th visit |
| 5. ◯ | Never |
| **B.6.** Was blood pressure measured and recorded at each visit? | |
| 1. ◯ | During 1st visit |
| 2. ◯ | During 2nd visit |
| 3. ◯ | During 3rd visit |
| 4. ◯ | During 4th visit |
| 5. ◯ | Never |
| **B.7.** Is the fetal heartbeats readings recorded? | |
| 1. ◯ | During 1st visit |
| 2. ◯ | During 2nd visit |
| 3. ◯ | During 3rd visit |
| 4. ◯ | During 4th visit |
| 5. ◯ | Never |
| **B.8.** Is proteinuria measured and recorded? | |
| 1. ◯ | During 1st visit |
| 2. ◯ | During 2nd visit |
| 3. ◯ | During 3rd visit |
| 4. ◯ | During 4th visit |
| 5. ◯ | Never |
| **B.9.** Are the results of Hemoglobin tests recorded for each visit? | |
| 1. ◯ | During 1st visit |
| 2. ◯ | During 2nd visit |
| 3. ◯ | During 3rd visit |
| 4. ◯ | During 4th visit |
| 5. ◯ | Never |
| **B.10.** Is Blood Sugar recorded? | |
| 1. ◯ | During 1st visit |
| 2. ◯ | During 2nd visit |
| 3. ◯ | During 3rd visit |
| 4. ◯ | During 4th visit |
| 5. ◯ | Never |
| **B.11.** Is number of pregnancies/Gravida (Number of previous pregnancies including the current pregnancy) recorded? | |
| 1. ◯ | Yes |
| 2. ◯ | No |
| **B.12.** Are the results of syphilis test recorded? | |
| 1. ◯ | Yes |

| | |
|---|---|
| 2. ◯ | No |
| **B.13.** Is provision of iron supplementation recorded? | |
| 1. ◯ | Yes |
| 2. ◯ | No |
| **B.14.** Is provision of malaria prophylaxis (Super net) recorded? | |
| 1. ◯ | Yes |
| 2. ◯ | No |
| **B.15.** Is HIV test recorded? | |
| 1. ◯ | Yes |
| 2. ◯ | No |
| **B.16.** Was tetanus Immunization given? | |
| 1. ◯ | Yes |
| 2. ◯ | No |
| 3. ◯ | No, she received it during previous pregnancy/ies |
| **B.17.** Is there any note on violence ongoing in the home? | |
| 1. ◯ | Yes |
| 2. ◯ | No |
| **B.18.** | Were Antihelmintic given |
| | Yes |
| | No |
