# Supplementary material for: Quality of antenatal care services in Rwanda: assessing practices of health care providers
Source: BMC Health Serv Res. 2018 Nov 19;18:865. doi: 10.1186/s12913-018-3694-5 (PMC6245627; doi:10.1186/s12913-018-3694-5)
Supplement: Supplementary file 2 — Table S1. Associations between health care providers’ characteristics and poor knowledge of conditions needing urgent assessment at a higher level of health care facility during pregnancy. (DOCX 62 kb) [file 12913_2018_3694_MOESM2_ESM.docx]

**Table S1 Associations between provider characteristics and poor knowledge of conditions needing urgent assessment during pregnancy (N=312).^‡^**

|  | **Poor knowledge of danger signs of pregnancy** | |
| --- | --- | --- |
| **Characteristic** | **Crude odds ratio (95% CI)** | **Adjusted odds ratio (95% CI)*** |
| **Age of providers** |  |  |
| 21-30 | 1 | 1 |
| 31-40 | 1.28(0.71, 2.33) | 1.34(0.69, 2.63) |
| ≥41 | 1.18(0.55, 2.55) | 1.33(0.56, 3.51) |
| **Sex** |  |  |
| Male | 1 | 1 |
| Female | 1.81(0.91, 3.58) | 1.67(0.81, 3.46) |
| **Profession** |  |  |
| Midwives |  | 1 |
| Nurses (Auxiliary, A2, A1, A0) | 1.38(0.49, 0.38) | 1.23(0.43, 3.51) |
| **Ever been invited to training** |  |  |
| Yes | 1 | 1 |
| No | 1.04(0.44, 2.42) | 1.12(0.44, 2.29) |
| **Years of experience in ANC services** |  |  |
| ≥4years | 1 | 1 |
| ≤3 years | 1.18(0.69, 2.02) | 1.32(0.72, 2.44) |
| **Consultation time (minutes)** |  |  |
| ≥16 | 1 | 1 |
| ≤15 | **2.25(1.29, 3.91)** | **2.14(1.21, 3.78)** |

**^‡^**Poor knowledge was defined as mentioning ≤2 conditions ; *All the variables in the table appeared in the same model
